# FLASH-RT for pulmonary protection: a comprehensive review of mechanisms, technological advances, and clinical translation

**DOI:** 10.3389/fonc.2025.1642745

**Published:** 2025-09-10

**Authors:** Yixue Wen, Xinlan Liu, Xiaohong Zhang, Li Long, Jing Feng, Zhen Zhang

**Affiliations:** ^1^ Breast Disease Center, Mianyang Central Hospital, School of Medicine, University of Electronic Science and Technology of China, Mianyang, China; ^2^ Departmant of Oncology, Mianyang Central Hospital, School of Medicine, University of Electronic Science and Technology of China, Mianyang, China; ^3^ Department of Oncology, Affiliated Hospital of North Sichuan Medical College, Nan Chong, Sichuan, China

**Keywords:** FLASH radiotherapy (FLASH-RT), pulmonary protection, mechanisms, technological advances, clinical translation

## Abstract

FLASH radiotherapy (FLASH-RT), characterized by ultra-high dose rates (>40 Gy/s), has demonstrated remarkable normal tissue-sparing effects in preclinical models while maintaining tumor control. This review specifically focuses on FLASH-mediated pulmonary protection, a critical concern in thoracic oncology. We critically evaluate proposed mechanisms—including oxygen depletion, radical recombination, mitochondrial preservation, DNA integrity maintenance, metabolic modulation, and immune reprogramming—with an emphasis on the strength and limitations of current evidence across *in vitro*, *in vivo*, and emerging clinical studies. Additionally, we summarize recent technological advances enabling clinical translation, such as FLASH-compatible beam modalities, real-time dosimetry, and motion management strategies. Unlike previous reviews, we integrate these mechanisms into a unified conceptual model and provide a structured comparison of evidence quality and contradictions. This work aims to clarify current controversies, highlight knowledge gaps, and guide future research and clinical trial design for FLASH-RT–based lung protection.

## 1 Introduction

Cancer is one of the leading causes of human mortality at present (1). Radiotherapy is currently one of the primary treatment modalities for malignant tumors. It is estimated that 50%-60% of cancer patients require radiotherapy either as a standalone intervention or in combination with other therapeutic strategies (2–6). Importantly, the number of patients requiring RT is expected to increase in the foreseeable future (7). The fundamental objective of radiotherapy lies in delivering the prescribed tumoricidal dose while minimizing radiation-induced damage to adjacent healthy tissues (2, 5, 8). Over recent decades, significant advancements have been achieved in radiotherapy delivery techniques, with modalities such as image-guided radiotherapy (IGRT), intensity-modulated radiotherapy (IMRT), and stereotactic radiotherapy (SRT) establishing radiotherapy as a paradigm of precision medicine (9–14). However, conventional radiotherapy (CONV-RT) is often constrained by the maximum tolerance dose of surrounding normal tissues, which limits its optimal antitumor efficacy (15–17). Radiation-induced lung injury (RILI), encompassing pneumonitis and fibrosis, remains a dose-limiting toxicity in thoracic radiotherapy, affecting 15–30% of patients (18–21). CONV-RT exacerbates RILI through prolonged oxidative stress and chronic inflammation (22–25).

FLASH-RT refers to a radiation therapy modality that utilizes ultra-high dose rate (UHDR) irradiation (>40 Gy/s, compared to CONV-RT dose rates typically <0.17 Gy/s) delivered within extremely short timeframes (generally <1 second) (26). FLASH-RT is a disruptive new technology in tumor radiotherapy, which is regarded as a significant technological advancement influencing tumor treatment and is one of the most popular research areas in radiotherapy in recent years. Compared with conventional dose rate radiotherapy, FLASH-RT has two major advantages: ①The treatment schedule can be significantly shortened, from several weeks (conventional fractionation) to only a few fractions, with each fraction delivered within milliseconds;②The enhanced sparing of normal tissues, which gives rise to the FLASH effect, is thought to result from a combination of mechanisms, including altered redox chemistry, transient hypoxia, preservation of DNA integrity, and modulation of immune and inflammatory responses. Moreover, by precisely decreasing the volume of the radiotherapy target area, the damage of radiation to adjacent critical structures can be minimized. What’s more, compared with CONV-RT, FLASH-RT has the same lethality to tumor tissues. Simply put, preclinical studies demonstrate that FLASH-RT preserves equivalent tumor control probability (TCP) compared to CONV-RT while significantly lowering normal tissue complication probability (NTCP), particularly in radiosensitive organs such as pulmonary tissues. This paradigm-shifting technology holds promise for optimizing the therapeutic ratio by simultaneously improving treatment efficiency, maintaining oncological efficacy, and mitigating radiation-related toxicities (27–31).

While several recent reviews have provided general overviews of FLASH-RT’s mechanisms and technological potential, they often lack an organ-specific perspective and provide limited discussion of emerging data published after 2023. In particular, the pulmonary protection conferred by FLASH-RT—a critical consideration for thoracic oncology—has not yet been comprehensively addressed. This review seeks to fill this gap by critically synthesizing the current understanding of FLASH-RT’s lung-specific radioprotective mechanisms, integrating recent preclinical and early-phase clinical studies, and identifying key challenges in clinical translation. We also discuss novel hypotheses and future research directions aimed at optimizing FLASH-RT for thoracic applications. In doing so, this review provides a timely and targeted update on the evolving landscape of FLASH-RT with a focus on pulmonary protection.

## 2 Mechanisms of FLASH-mediated lung protection

**Figure 1:**
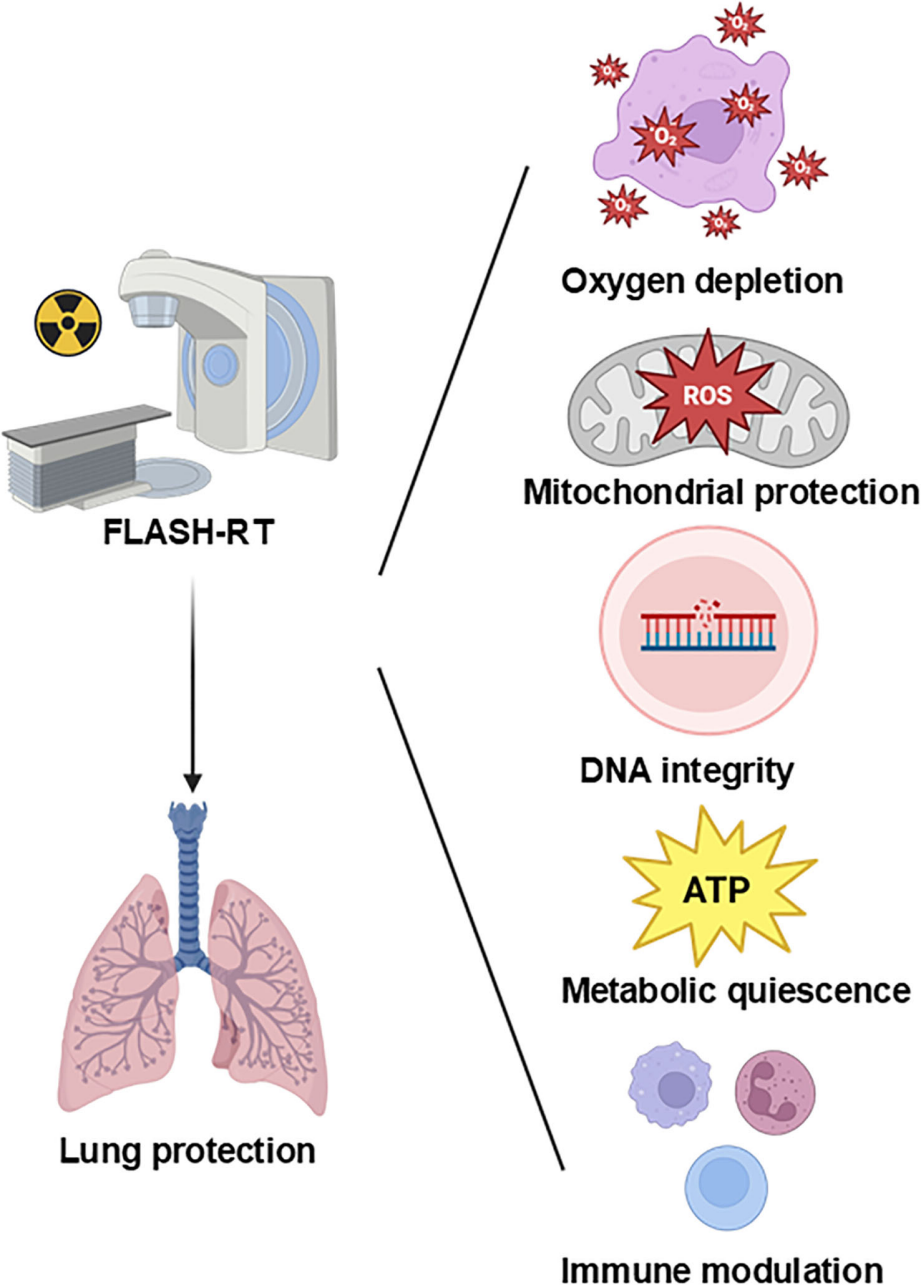
Proposed mechanisms underlying the pulmonary protective effects of FLASH radiotherapy.

Multiple hypotheses have been proposed to explain the FLASH effect, including oxygen depletion, redox modulation, mitochondrial protection, DNA integrity preservation, and immune modulation. Most supporting evidence comes from preclinical studies, particularly *in vitro* models and murine systems. However, the extrapolation of these findings to humans remains limited by differences in tissue complexity, tumor microenvironment, and beam parameters. Below, we critically evaluate each mechanism, the strength and limitations of available data, and unresolved controversies (**Figure 1**).

### 2.1 Oxygen depletion hypothesis

**Figure 2:**
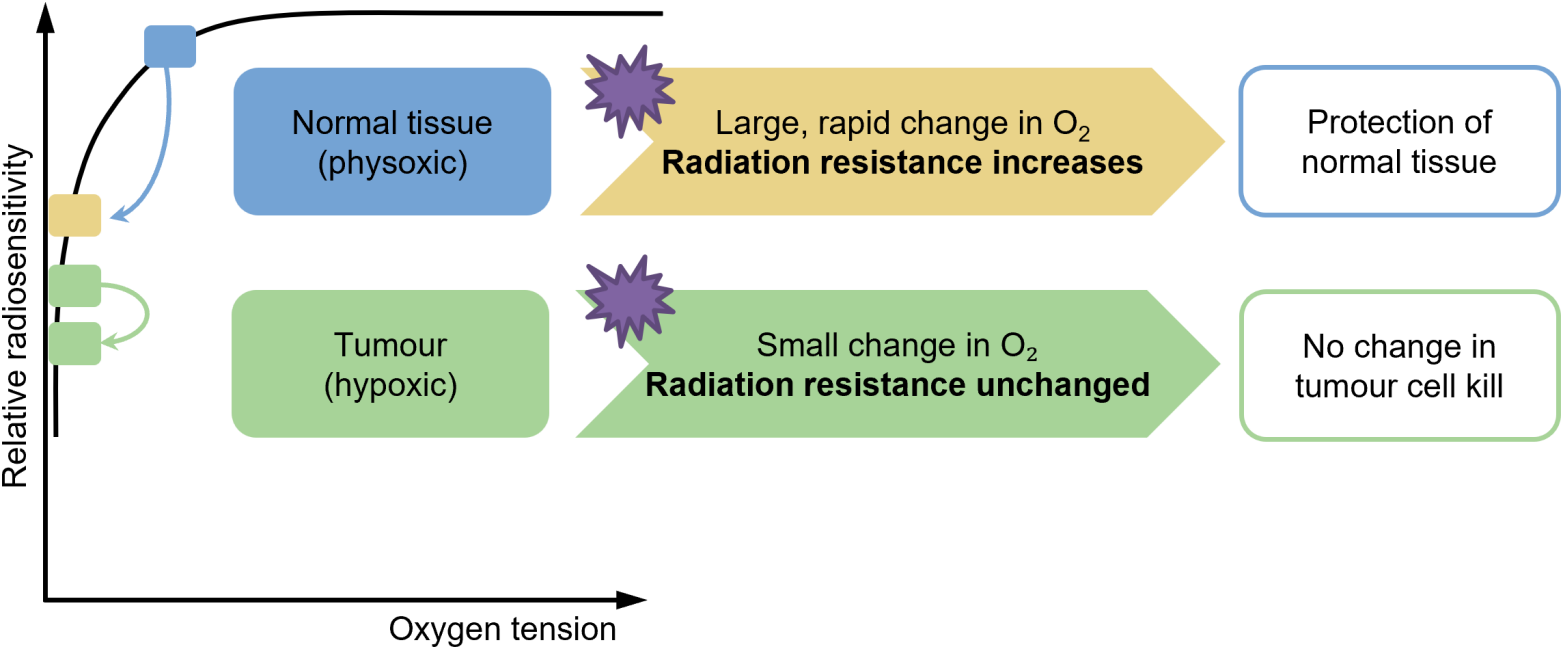
Illustration of oxygen depletion in the FLASH effect.

It is well known that oxygen acts as a radiosensitizing agent, and the presence of oxygen when irradiated can increase the radioactive effect (32–34). The oxygen depletion hypothesis (**Figure 2**) posits that the ultra-high dose rate radiation therapy mitigates normal tissue toxicity, particularly in the lung, by transiently reducing intracellular oxygen tension during irradiation, thereby limiting the formation of radiation-induced reactive oxygen species (ROS) (35–38). CONV-RT generates ROS via the radiolysis of water, which is potentiated by molecular oxygen (O_2_), leading to DNA damage and subsequent cellular apoptosis in both tumor and normal tissues. In contrast, FLASH-RT delivers radiation at dose rates exceeding 40 Gy/s, which is hypothesized to rapidly deplete local oxygen reserves within milliseconds, creating a transient hypoxic microenvironment. This acute oxygen depletion attenuates the radiosensitizing effects of O_2_ in normal lung parenchyma, while tumor cells, often residing in chronically hypoxic niches, remain vulnerable to radiation-induced damage due to their impaired repair mechanisms (39–41). Experimental studies in murine models have demonstrated that FLASH-RT significantly reduces pulmonary inflammation, fibrosis, and oxidative stress markers compared to conventional dose rates, aligning with the oxygen depletion paradigm (36). Furthermore, *in-vitro* assays using lung epithelial cells under controlled hypoxic conditions replicate the radioprotective effects observed with FLASH, supporting the critical role of oxygen dynamics. However, the precise spatiotemporal resolution of oxygen consumption during FLASH and its differential impact on tumor versus normal tissue microenvironments require further elucidation (42). Current evidence underscores the oxygen depletion hypothesis as a pivotal mechanism underlying FLASH-mediated lung protection, offering a promising avenue to enhance the therapeutic index of radiotherapy (42, 43). However, the oxygen depletion hypothesis remains controversial. While early studies demonstrated significant oxygen consumption under ultra-high dose rates (44), more recent findings suggest that oxygen depletion is highly dependent on beam type, tissue oxygenation, and model system used (45). Additionally, most evidence derives from *in vitro* or animal studies; human data remain scarce. Therefore, oxygen depletion is likely one of several synergistic mechanisms rather than the sole driver of the FLASH effect.

### 2.2 Free radical interaction hypothesis

Free radicals are highly reactive molecules. They possess unpaired electrons in their outer orbitals, which makes them extremely unstable. Once formed within cells, these free radicals can readily initiate a cascade of chemical reactions. For instance, they can react with various cellular components such as DNA, proteins, and lipids. When they react with DNA, they may cause strand breaks, base modifications, and cross-links, all of which can potentially lead to cell damage or even cell death (46, 47).

**Figure 3:**
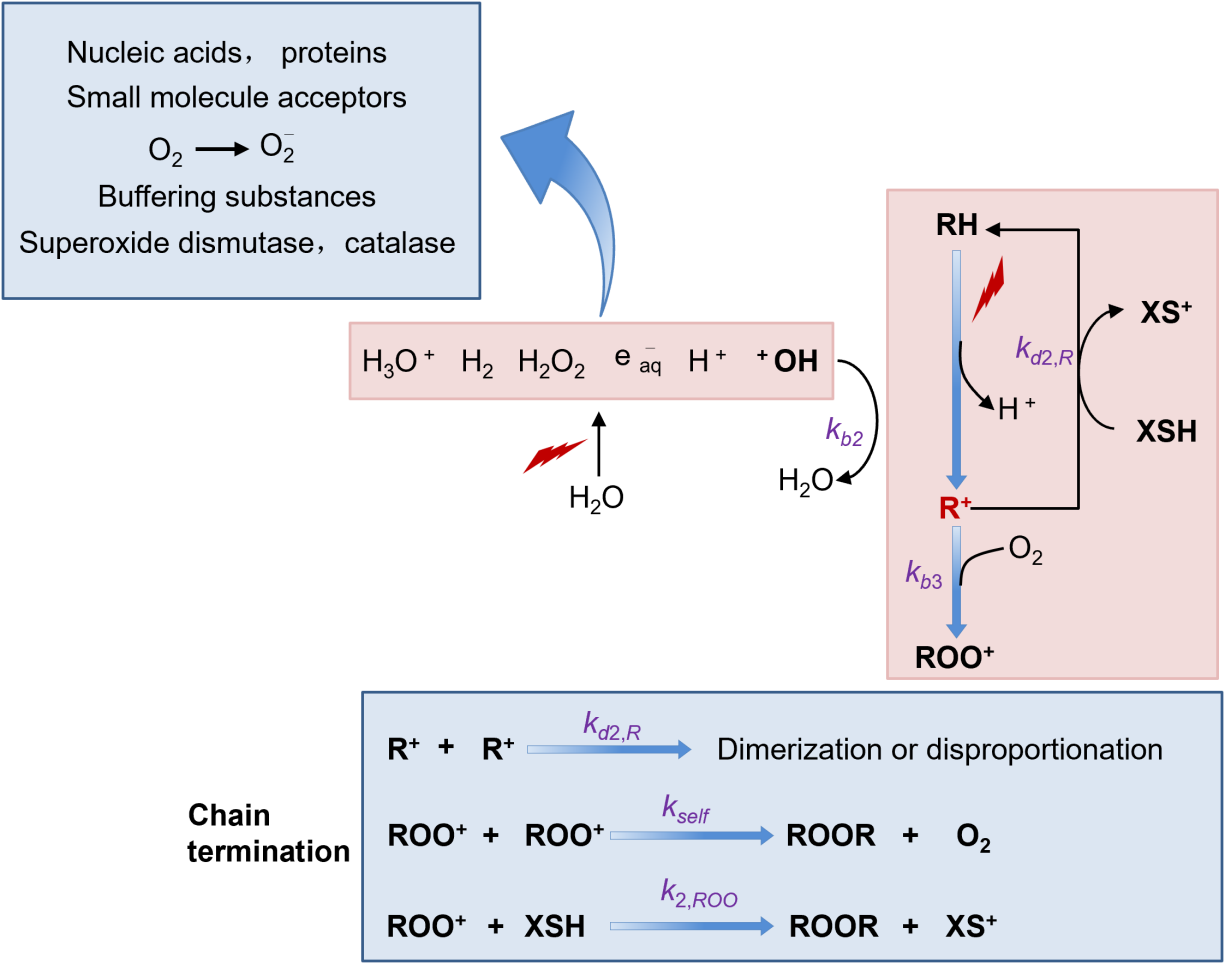
Schematic illustration of free radical interactions relevant to the FLASH effect.

In the context of FLASH-RT, which is characterized by high dose-rates, an interesting phenomenon occurs with free radicals. At these high dose-rates, a large number of free radicals are generated instantaneously, resulting in a transiently high concentration. Under such conditions, there is an increased probability for free radicals to react with each other (36, 48). This intermolecular reaction between free radicals can lead to the formation of more stable molecules. As a consequence, the overall number of free radicals available to cause damage to cells is reduced. However, free radicals also have another reaction pathway. They can react with molecular oxygen to form reactive oxygen species (ROS), which include superoxide anion (O_2_^-^), hydrogen peroxide (H_2_O_2_), and hydroxyl radical (·OH). These ROS are also highly reactive and can cause significant damage to cells, similar to free radicals themselves (38, 46)(**Figure 3**).

Numerous studies have demonstrated that there are distinct differences between normal cells and cancer cells in terms of their ability to scavenge ROS (49). Normal cells are equipped with an efficient antioxidant defense system. They contain various antioxidant enzymes such as superoxide dismutase (SOD), catalase (CAT), and glutathione peroxidase (GPx). These enzymes work in concert to neutralize ROS and maintain cellular redox balance. In contrast, tumor cells often have an over-abundance of reactive metal ions, such as iron and copper. These metal ions can participate in Fenton-like reactions, further generating more ROS. Additionally, the antioxidant enzyme systems in tumor cells are relatively weaker compared to normal cells (50). As a result, tumor cells are less efficient at clearing ROS. This difference in ROS scavenging ability between normal and tumor cells may provide an explanation for the observed effects of FLASH-RT. FLASH-RT seems to have a protective effect on normal tissues. The reduced damage to normal tissues may be attributed to the self-quenching of free radicals at high dose-rates, which in turn leads to a decrease in ROS production. On the other hand, the anti-tumor effect of FLASH-RT on tumors does not show a significant difference compared to traditional radiotherapy. This is because tumor cells, with their poor ROS-scavenging ability, are still vulnerable to the remaining ROS even under FLASH-RT conditions (51, 52).

Both *in-vitro* and *in-vivo* studies have provided evidence to support these concepts. *In-vitro* studies have shown that after FLASH-RT irradiation, the levels of lipid peroxides, which are markers of oxidative stress, and ROS content in normal cell lines are decreased (41, 45, 50, 51, 53, 54). *In-vitro* cell experiments have shown that under the condition of FLASH-RT, the free radical level in lung cells rapidly increases and then quickly decreases within a short period of time, and the activity of antioxidant enzymes and the content of endogenous antioxidant substances in the cells increase significantly. In animal experiments, after FLASH-RT of the lungs, by detecting the oxidative stress indexes in lung tissues, it was found that the content of lipid peroxidation products was lower than that in the CONV-RT group, and the expression of antioxidant enzyme genes and proteins was up-regulated (55).

FLASH-RT appears to preferentially protect mitochondrial function in normal cells. This may reflect intrinsic differences in mitochondrial metabolism between normal and tumor cells. Tumors often exhibit the Warburg effect, relying primarily on glycolysis with altered mitochondrial dynamics, which could render them less vulnerable to mitochondrial preservation by FLASH. Recent proteomic and metabolic studies show FLASH-RT downregulates oxidative phosphorylation markers selectively in healthy lung tissue while maintaining tumor suppression, suggesting a cell type–dependent mitochondrial response (53). However, it should be noted that there are significant differences between *in-vitro* and *in-vivo* environments. *In-vitro* systems lack the complexity of the *in-vivo* microenvironment, including factors such as blood flow, immune cell interactions, and tissue-specific physiological conditions. Therefore, more *in-vivo* studies are urgently needed to comprehensively understand the different mechanisms of FLASH-RT and CONV-RT with respect to ROS generation, scavenging, and their impact on normal and tumor tissues (41). regulated (55).

### 2.3 Mitochondrial hypothesis

Mitochondria, as the core of cellular metabolism, generate energy through the process of oxidative phosphorylation, converting chemical energy into adenosine triphosphate (ATP). Meanwhile, they regulate cell death and signal transduction (56). Mitochondrial reactive oxygen species (mtROS) are the main source of intracellular reactive oxygen species and are involved in redox metabolism and apoptosis (57–59). Radiation can directly or indirectly damage mitochondrial DNA (mtDNA), leading to mitochondrial dysfunction and apoptosis (60–63). Compared with CONV-RT, FLASH-RT can protect the mitochondrial function of normal cells, reduce mitochondrial damage and the production of reactive oxygen species, and regulate the expression of mitochondrial-related proteins, thereby reducing the proportion of cell apoptosis and necrosis (64, 65). This protective effect may be achieved by reducing mitochondrial damage and mtROS imbalance, which helps maintain the homeostasis of normal cells. In contrast, tumor cells are more sensitive to changes in reactive oxygen species. FLASH-RT may cause the death of tumor cells by increasing mtROS, so that the tumor control effect is similar to that of CONV-RT, providing a new perspective on the mechanism of FLASH-RT (41, 48).

**Figure 4:**
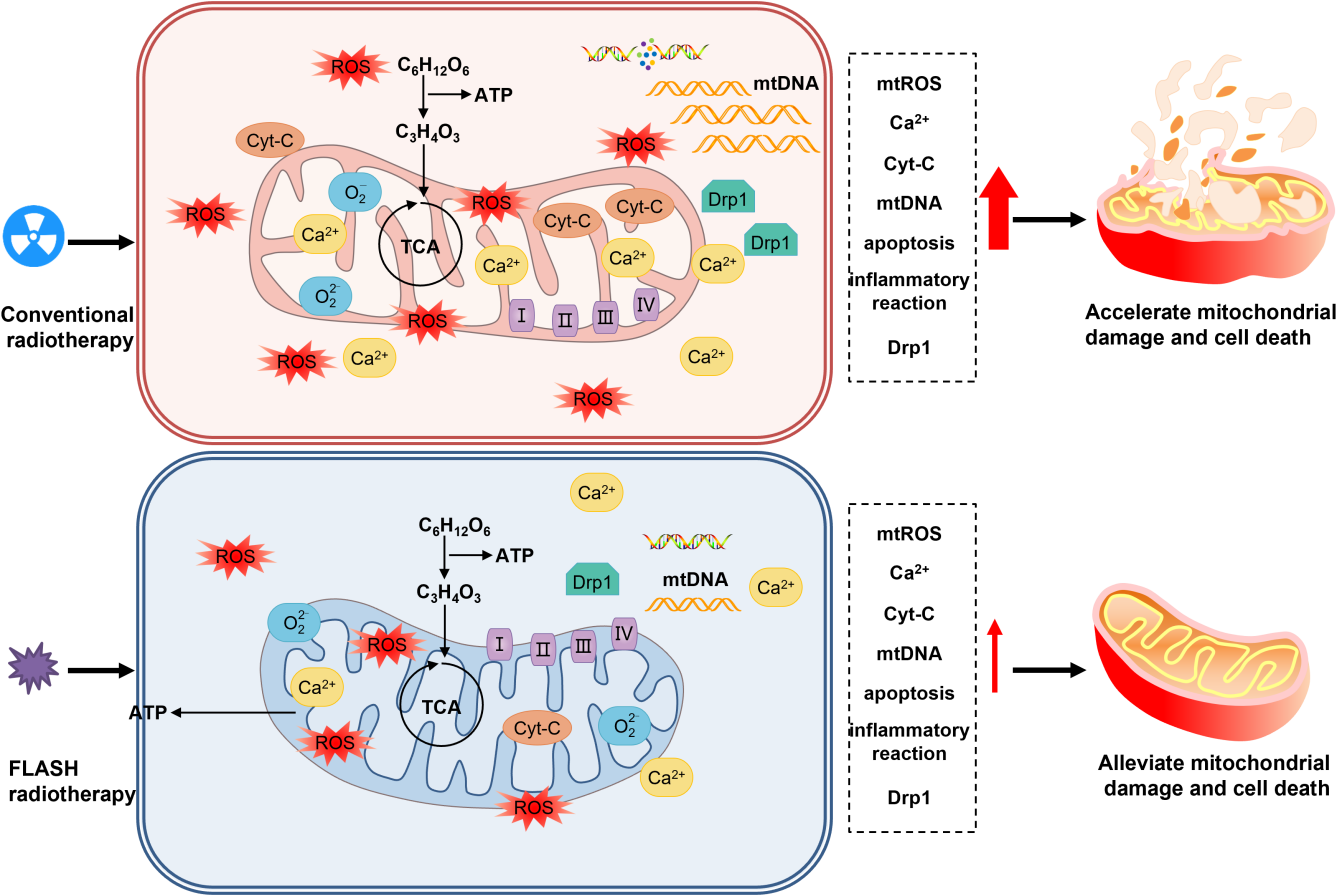
Illustration of mitochondrial in the FLASH effect.

Many studies have provided support for the mitochondrial metabolism hypothesis of FLASH-RT (64, 65)(**Figure 4**). At the same time, studies on tumor cells have found that after FLASH-RT, the ROS level in tumor cells increases significantly, the mitochondrial function is impaired, and the cell apoptosis rate increases (48).

Although the mitochondrial metabolism hypothesis provides an important theoretical framework for the protective mechanism of FLASH-RT against lung injury, there are still some unresolved issues. For example, the specific molecular mechanisms and signaling pathways of mitochondrial metabolic changes induced by FLASH-RT are not fully understood, and it remains to be further investigated whether there are differences in the mitochondrial responses of different types of lung cells (such as alveolar epithelial cells, pulmonary vascular endothelial cells, etc.) to FLASH-RT. In the future, more in-depth basic research and clinical trials are needed to verify and refine this hypothesis, providing a more solid theoretical foundation and practical guidance for the widespread clinical application of FLASH-RT. In conclusion, the mitochondrial metabolism hypothesis offers a crucial perspective for understanding the protective mechanism of FLASH-RT against lung injury, and it is expected to drive the further development and optimization of tumor radiotherapy techniques.

### 2.4 DNA integrity hypothesis

The classical target theory posits that DNA serves as the primary target of ionizing radiation. Specifically, the double-strand breaks in DNA are regarded as the main cause of cell mutation and death, which pose a serious threat to genomic stability. In the complex process of radiation interaction with biological systems, the integrity of DNA is of utmost importance. Any damage to DNA can trigger a series of cellular responses, and double-strand breaks are particularly critical as they can disrupt the normal genetic information transmission and lead to various adverse consequences (66–70).

**Figure 5:**
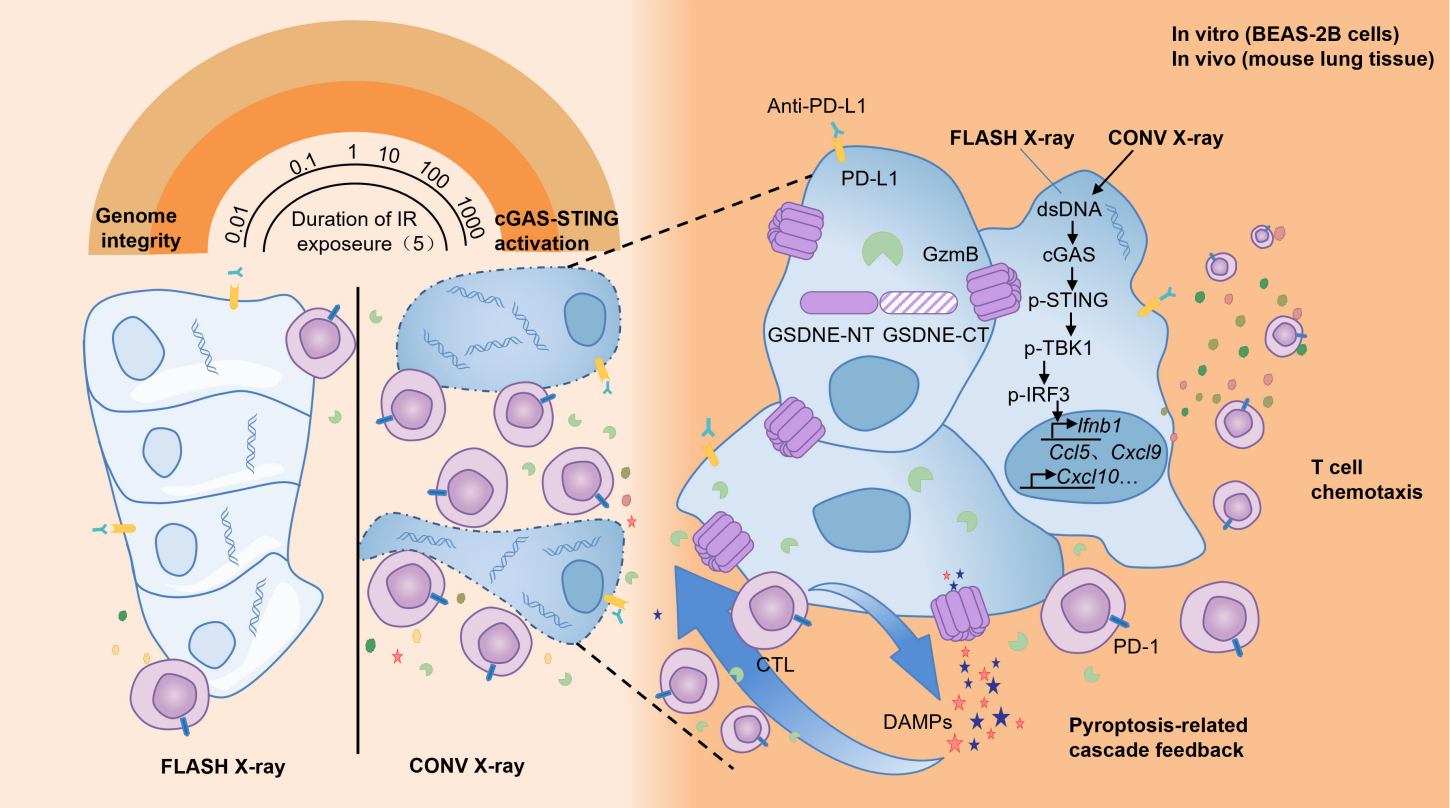
Illustration of DNA integrity in the F6LASH effect.

Moreover, high linear energy transfer (LET) radiation, such as protons, has a greater propensity to induce complex and difficult-to-repair damages. High LET radiation deposits energy more densely along its track, causing more clustered lesions in DNA, which are far more challenging for the cell’s repair mechanisms to handle compared to damages induced by low LET radiation (71–74).Therefore, understanding the DNA damage response following FLASH-RT is of great significance for comprehending the FLASH effect (**Figure 5**). Ohsawa et al. dissolved pBR322 plasmid DNA in 1×TE buffer and exposed it to 27.5 MeV protons. They found that, in comparison with the conventional dose-rate proton therapy (COVN-PT, at a dose rate of 0.05 Gy/s), the FLASH-RT group (at a dose rate of 40 Gy/s) had a significant reduction in single-strand DNA breaks (75). In the context of cellular biology, single-strand DNA breaks are relatively easier to repair in living cells (76, 77). Given this, it is reasonable to postulate that FLASH irradiation can reduce non-lethal damages associated with late-effects, such as cell senescence and genomic instability, thereby protecting normal tissues. This reduction in non-lethal damages may be attributed to the unique physical and biological characteristics of FLASH irradiation, which could potentially modulate the cellular response to radiation damage in a way that is beneficial for normal tissue sparing.

Many *in-vitro* cell experiments have shown that FLASH-RT causes less DNA damage to cells. For example, in human lung epithelial cell lines, when comparing CONV-RT and FLASH-RT, it was found that the number of γ-H2AX (a marker of DNA double-strand breaks) foci formed in the FLASH-RT group was significantly reduced, indicating that FLASH-RT induced fewer DSBs (42, 74). In addition, through the comet assay to detect the degree of DNA damage, it was also confirmed that the migration length of cellular DNA under FLASH-RT was shorter, that is, the degree of DNA damage was lower. In the mouse model of lung radiation injury, compared with mice receiving CONV-RT, the pathological changes in the lung tissue of mice receiving FLASH-RT were significantly alleviated. Histological analysis revealed that the degree of fibrosis in the lung tissue of the FLASH-RT group was lower, and the alveolar structure suffered less destruction. Further research demonstrated that the expression of genes associated with DNA damage repair in the lung tissue cells of the FLASH-RT group tended more towards normal levels, indicating that DNA integrity was better preserved, thereby reducing lung injury (42, 78).

Although no clinical studies have yet confirmed the protective effects of FLASH-RT on lung tissue, numerous preclinical studies have provided supporting evidence. In animal models, FLASH-RT has been shown to reduce radiation-induced lung injuries, such as pneumonitis and fibrosis, compared to CONV-RT. These protective effects are associated with decreased inflammation, reduced oxidative stress, and better preservation of tissue architecture, as demonstrated by histological analysis and molecular markers (66, 79, 80).

While the preservation of DNA integrity is one of the key hypotheses explaining the lung-sparing effect of FLASH-RT, several challenges remain (81). For example, different pulmonary cell types and tissue structures may exhibit heterogeneous responses to ultra-high dose rate irradiation. Optimizing the parameters of FLASH-RT to effectively preserve DNA integrity across various lung compartments still requires extensive investigation. Furthermore, the specific molecular mechanisms by which FLASH-RT influences DNA damage recognition and repair processes remain largely unclear, necessitating further in-depth mechanistic research.

### 2.5 Metabolic quiescence hypothesis

Cancer cells exhibit a unique metabolic phenotype whereby they preferentially utilize aerobic glycolysis for energy production, even under normoxic conditions—a phenomenon known as the Warburg effect (82–86). During CONV-RT, ionizing radiation induces mitochondrial dysfunction, compromising oxidative phosphorylation (OXPHOS) in both tumor and normal tissues. This disruption leads to excessive production of reactive oxygen species (ROS), impaired ATP generation, and ultimately cell death (60–63).

**Figure 6:**
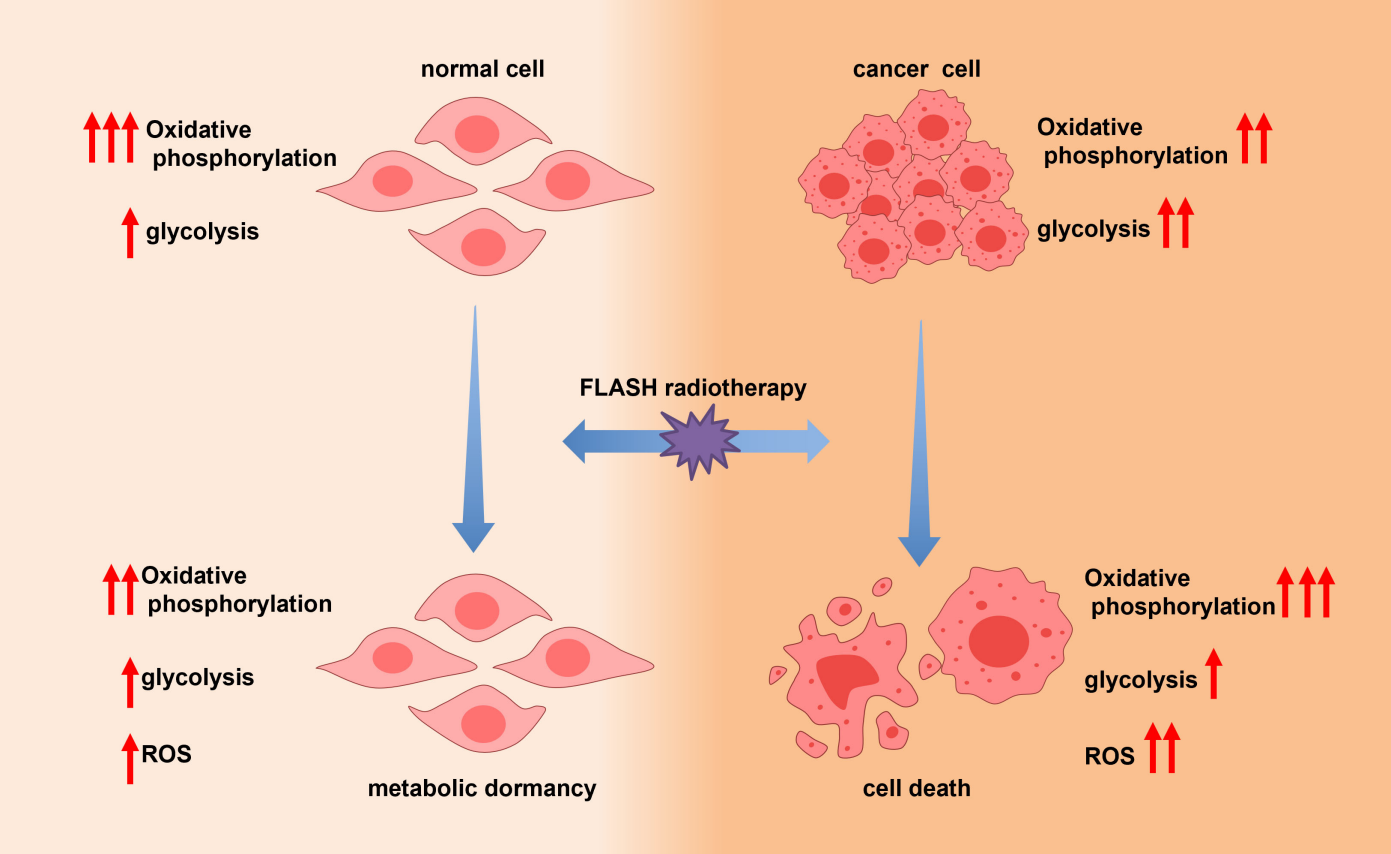
Illustration of metabolic quiescence in the FLASH effect.

In contrast, FLASH-RT exhibits different metabolic effects (40, 87–89). Emerging evidence suggests that FLASH-RT may preserve mitochondrial integrity and OXPHOS activity in normal cells. Moreover, normal lung cells may enter a state of metabolic quiescence—a transient, energy-conserving condition characterized by suppressed OXPHOS and biosynthetic activity, functionally resembling cellular dormancy. In this state, cells reduce ATP consumption by downregulating non-essential protein synthesis and decreasing the activity of energy-intensive ion pumps, thereby conserving metabolic resources and enhancing resistance to stress. Although real-time measurement of ATP levels and mitochondrial function during FLASH exposure remains technically challenging, preclinical studies support the notion that metabolic quiescence may serve as a protective mechanism against hypoxia, radiation, and other cytotoxic insults. This metabolic adaptation may underlie the observed tissue-sparing effects of FLASH-RT, particularly in highly sensitive organs such as the lungs (**Figure 6**).

### 2.6 Immune modulation

**Figure 7:**
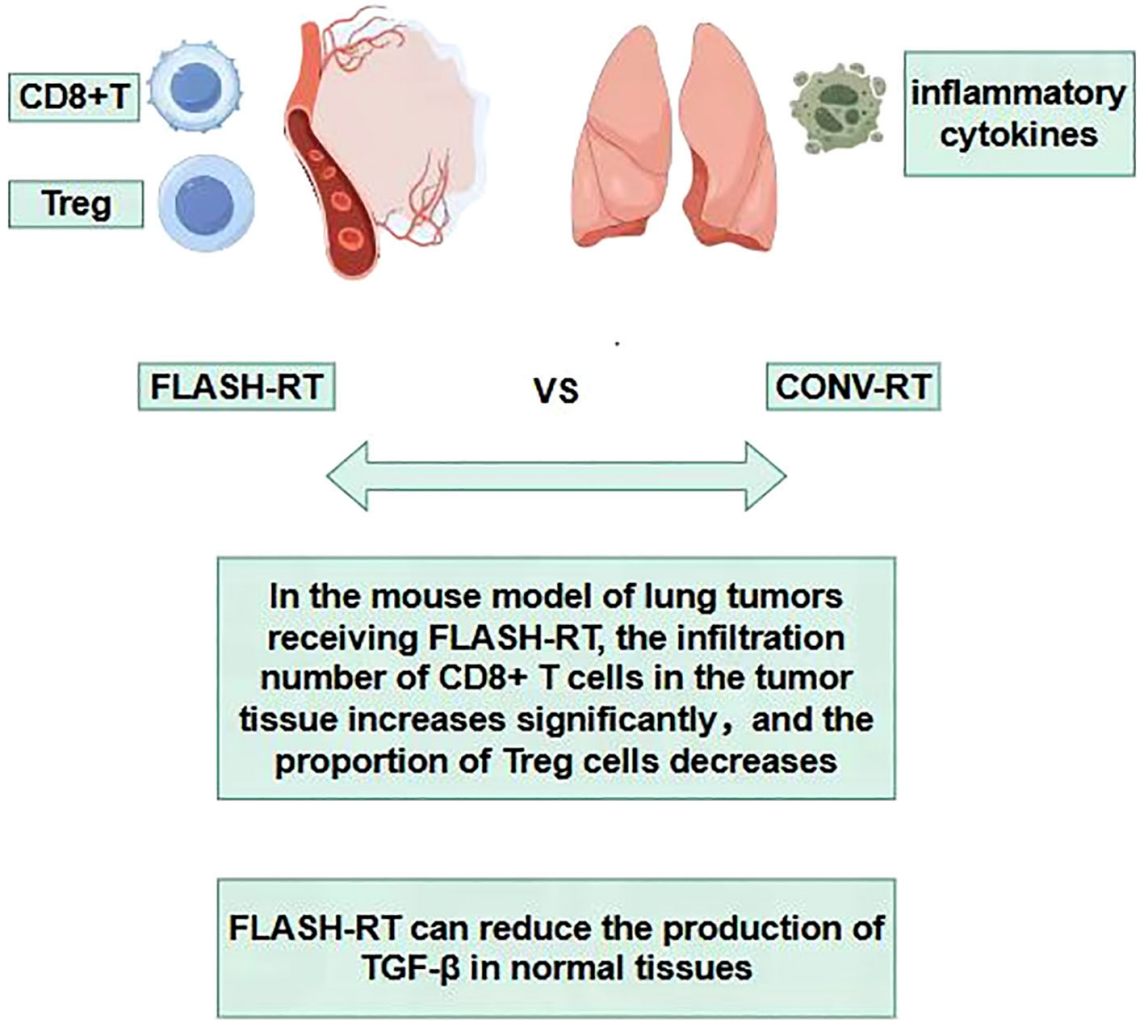
Immune hypothesis.

During the process of traditional CONV-RT, radiation can damage the immune cells in the circulating blood, reducing the immune function of the body (90–94). The sparing effect of circulating immune cells diminishes the destruction of the body’s immune system, helps the repair of damaged tissue cells and thus reduces the level of tissue damage. Studies have found that in experimental animals receiving FLASH-RT, the number and activity of immune cells such as lymphocytes in the peripheral blood decrease significantly less compared with those in the CONV-RT group after radiotherapy (95). This indicates that FLASH-RT has a protective effect on circulating immune cells (96–98), which helps maintain the stability of the overall immune function of the body. Some studies have found that in the mouse model of lung tumors receiving FLASH-RT, the infiltration number of CD8^+^ T cells in the tumor tissue increases significantly, and at the same time, the proportion of Treg cells decreases (72, 95, 99, 100). In the lung tissue, FLASH-RT may promote the infiltration of immune cells into the tumor tissue by changing the tumor microenvironment (95). This change in the pattern of immune cell infiltration is conducive to enhancing the immune surveillance and killing effect of the body on tumor cells, and reducing the immune damage to normal lung tissue(**Figure 7**). The comparisons of characteristics of FLASH-RT and CONV-RT were concluded in **Table 1**.

**Table 1:** Comparison of characteristics of FLASH-RT and CONV-RT.

Comparison Items	FLASH-RT	CONV-RT
Mean Dose Rate	≥40Gy/s	≤1 Gy/min
Treatment Time	< 200 ms	> 1 min
Dose Delivery	High dose in a single fraction	Low dose in a single fraction
Tumor Control	Similar antitumor effect as CONV-RT (45)	Effective tumor killing
Normal Tissue Sparing	Reduce damage to healthy tissues	Acute and late damage to healthy tissues
Advantages	Has optimal dosimetric and geometric parameters recommended by expert consensus	Reduces acute toxicity and delays late toxicity
	Prescribed doses with high evidence-based medical evidence	Tumor control effects similar to conventional dose rates
	Proven segmentation options	Reduced treatment duration
Disadvantages	Less effective radiotherapy for some tumors with poor sensitivity	The optimal dose rate for clinical application is not yet known
	Inevitably causes organ-threatening damage	The effectiveness of treatments for different diseases is not yet clear
	Longer treatment period	Equipment is expensive and may increase the cost of patient care
Current Limitations	Requires ultra-high dose rate equipment, motion-adaptive planning, and real-time dosimetry; limited clinical protocols and regulatory standards	Lower tissue sparing; longer treatment time; risk of cumulative toxicity

Traditional radiotherapy can trigger an inflammatory response in the lung tissue, leading to the massive release of inflammatory cytokines such as Transforming growth factor-β (TGF-β), tumor necrosis factor-α (TNF-α), interleukin-6 (IL-6), etc. These inflammatory cytokines will recruit immune cells to the lung tissue, causing immune-related lung injuries, such as radiation pneumonitis (101, 102). Among them, transforming growth factor-β (TGF-β) is a multifunctional cytokine that plays an important role in regulating the immune system and tumor growth (48, 103). After exposure to ionizing radiation, the increase in TGF-β has been proven to have side effects on different normal tissues, such as the induction of fibrosis (103). Studies have found that compared with CONV-RT, FLASH-RT can reduce the production of TGF-β in normal tissues, including mouse lungs (36), mouse skin (104–106), canine skin (105), and human lung fibroblasts (67). In addition, FLASH-IR was also proven to induce variations in other cytokines compared with CONV-IR, such as Cxcl-1, G-CSF, GM-CSF, IL-1β, IL-4, IL-6, IL-10, TNF-α, etc. These may all be the reasons why FLASH-RT reduces lung injury (104, 107, 108).

Regarding the immunological hypothesis, there are currently also some studies with negative results (109, 110). Moreover, there are still many unknowns and questions that require further investigation. For instance, the exact molecular mechanisms of immunomodulation by FLASH-RT and the specific roles of different immune cell subsets in protecting against lung injury. In the future, more in-depth basic research and large-scale clinical studies are needed to further verify and improve this hypothesis, providing a solid theoretical basis for the widespread application of FLASH-RT in lung cancer and other diseases that require thoracic radiotherapy. Nevertheless, not all studies have observed consistent immune responses to FLASH. Some preclinical experiments report minimal activation of innate or adaptive immunity, suggesting possible dose-dependency or tumor-type specificity (111). Furthermore, the paradox of lymphocyte sparing versus enhanced CD8^+^ T-cell infiltration remains unresolved and requires mechanistic clarification. Ongoing trials combining FLASH-RT with immune checkpoint inhibitors may shed light on these immune dynamics.

## 3 Technological innovations in flash delivery for lung applications

### 3.1 Beam modalities

In the context of FLASH radiotherapy for lung applications, a variety of beam modalities are being explored.

Electron beams have been among the first to be investigated for FLASH delivery (36, 80, 112–114). Their relatively shallow penetration depth makes them suitable for treating superficial lung tumors or lesions close to the body surface. For instance, in pre-clinical studies, electron FLASH has shown promising results in sparing normal lung tissue while effectively targeting tumors (67). A study by Fouillade et al. demonstrated that when electron FLASH was applied to a murine model with lung tumors, the normal lung parenchyma showed significantly less damage compared to CONV-RT (42). The high-energy electrons deposit energy rapidly within a short range, allowing for a sharp dose fall-off beyond the target volume, which is crucial for protecting adjacent healthy lung tissue.

Proton beams are also emerging as a viable option for FLASH radiotherapy in the lungs. Protons offer the advantage of a Bragg peak, where the majority of the energy is deposited at a specific depth, followed by a rapid dose decrease (43) This characteristic can be exploited in FLASH proton radiotherapy (FLASH-PT) to precisely target lung tumors while minimizing the dose to surrounding normal structures. However, the clinical implementation of proton FLASH faces significant challenges. A major bottleneck is the target conversion inefficiency in current cyclotron-based systems, which rely on energy degraders to reduce beam energy for clinical use. This process results in substantial proton losses, reduced dose rates, and excess heat generation, thereby limiting their FLASH capability. Additionally, achieving uniform ultra-high dose rates in deep-seated lung tumors is further complicated by technical constraints in beam scanning, energy modulation, and timing synchronization.

Recent technological breakthroughs have begun to address these barriers. Synchrotron-based proton sources offer improved control over beam energy without the need for energy degraders, enabling more efficient UHDR delivery. Moreover, novel approaches like Bragg peak FLASH, which deliver FLASH dose at the distal end of the proton path, are being explored for maximizing tumor selectivity and normal tissue sparing. Early feasibility studies suggest that these advances could significantly expand the scope of proton FLASH, particularly for thoracic and deep-tissue malignancies (115).

Photon beams, which are widely used in traditional radiotherapy, are also being adapted for FLASH delivery (116, 117). However, achieving ultra-high dose rates with photons poses unique challenges due to the nature of photon-matter interactions. Despite these challenges, researchers have developed innovative techniques to generate high-intensity photon beams for FLASH (116). For example, synchrotron-based photon sources can produce extremely high-dose-rate photon beams (118). These sources can deliver a large number of photons in a short time, enabling FLASH-like delivery. A study carried out by Montay-Gruel et al. revealed that the FLASH effect could be achieved in normal brain tissue using x-rays delivered at an instantaneous dose rate of 12,000 Gy/s (with a mean dose rate of 37 Gy/s). This partially replicated the outcomes previously obtained with 6 MeV electrons (116, 119).

### 3.2 Dosimetry

Accurate dosimetry is of utmost importance in FLASH radiotherapy for lung applications. The ultra-high dose rates and short delivery times in FLASH present new challenges for dosimetry systems. Traditional dosimeters, which are designed for CONV-RT, may not accurately measure the dose in FLASH conditions (120). Moreover, the absence of standardized UHDR-specific dosimetric tools and protocols remains a critical bottleneck for reliable clinical implementation.

For electron FLASH, ionization chambers need to be carefully calibrated to account for the high-dose-rate effects. The collection efficiency of charges in ionization chambers can be affected by the ultra-high dose rates, leading to inaccurate dose measurements (121). To mitigate this, improved parallel-plate ionization chamber designs with optimized electrode materials and geometries have been developed to enhance signal accuracy (122). However, standardization of real-time correction protocols across devices is still lacking.

For proton FLASH dosimetry, the Bragg peak profile and depth-dependent energy deposition require specialized techniques (115, 123–126). Monte Carlo simulations are often used to accurately predict the dose distribution in proton FLASH treatments (127–130). Complementing simulations, diamond detectors are increasingly investigated for their nanosecond-scale response, tissue equivalence, and radiation hardness, making them ideal for UHDR dose monitoring in proton beams (131–134). These detectors offer potential for integration into real-time feedback dosimetry systems, though clinical translation will require harmonized calibration standards and validation protocols. The summary of FLASH-RT in the thorax experiments was concluded in **Table 2**.

**Table 2:** Summary of Flash-RT in the thorax experiments.

Model	Radiation Source	Energy (MeV)	Dose (Gy)	Dose Rate (Gy/s)	Reference
Mouse (thorax)	Electrons	4.6	15.17	40	(35)
Mouse (thorax)	Electrons	20	14	70 - 210	(135)
Mouse (thorax)	Electrons	4.5	17	——	(42)
Mouse (thorax)	Protons	——	15, 17.5, 20	40	(136)
Mouse (thorax)	Protons	——	18	40	(137)
Mouse (thorax)	X-rays	0.32	15	352.1	(67)

For photon FLASH, the accurate measurement of high-energy photon fluence within sub-second delivery windows remain challenging. Traditional tools such as film dosimeters are limited by delayed response times and saturation effects (138). In contrast, solid-state dosimeters like metal-oxide-semiconductor field-effect transistor (MOSFET) sensors are under active evaluation for their fast response and real-time readout capabilities (116, 139–141). Despite their promise, MOSFETs still require careful dose-rate and energy spectrum calibration under FLASH-specific conditions. In summary, while several detector technologies—including advanced ionization chambers, diamond detectors, and MOSFETs—are being tailored for FLASH dosimetry, the field still lacks standardized tools and real-time calibration protocols for consistent and accurate UHDR measurements. Future efforts should prioritize the development of validated, universally accepted UHDR dosimetry frameworks to ensure clinical reproducibility and safety.

### 3.3 Treatment planning

Treatment planning in FLASH radiotherapy for lung applications is a complex process that requires careful consideration of the unique characteristics of FLASH delivery. The ultra-high dose rates and short treatment times in FLASH can affect the dose distribution and the biological response of the tissue (79).

In FLASH treatment planning, accurate patient anatomy modeling is essential. Computed tomography (CT) scans are commonly used to obtain the patient’s anatomical information. However, in the context of FLASH, the CT images need to be acquired with high temporal and spatial resolution to accurately capture the position of the lung tumors and the surrounding normal structures (142). This is because the lung is a highly mobile organ, and respiratory motion can cause significant changes in the position of the tumor during the short FLASH treatment time (143, 144).

The use of four-dimensional CT (4D-CT) has become increasingly important in CONV-RT treatment planning for the lungs. 4D-CT allows for the visualization of the lung motion over the respiratory cycle. By analyzing the 4D-CT data, the treatment planner can determine the internal target volume (ITV) that takes into account the movement of the tumor during respiration (145, 146). In the future, it is possible that we will be able to more accurately deliver the FLASH radiation dose directly to the tumor, utilizing 4D-CT technology. This advancement could potentially spare more of the healthy lung tissue, reducing side effects and improving patient outcomes.

In addition to accurate anatomy modeling, the biological effects of FLASH need to be incorporated into the treatment planning. The ultra-high dose rates in FLASH can result in different biological responses compared to conventional radiotherapy (26, 31, 43). For example, FLASH has been shown to reduce the radiation-induced damage to normal tissue by modulating the immune response and reducing the production of reactive oxygen species (35–38). Accordingly, treatment planning systems are evolving to incorporate dose-rate-dependent biological response models (147, 148). One notable innovation is the Simultaneous Dose and Dose Rate Optimization (SDDRO) algorithm proposed by Hao Gao et al (149). This approach jointly optimizes both spatial dose distribution and temporal dose rate to maximize FLASH effect coverage while minimizing normal tissue toxicity. Compared with intensity-modulated proton therapy (IMPT), SDDRO significantly improves FLASH-dose rate coverage, as demonstrated by favorable dose rate volume histograms and increased normal tissue sparing.

However, despite these promising advances, SDDRO’s clinical scalability to thoracic targets remains to be fully validated. Lung tumors often exhibit large inter-patient variability in motion amplitude, anatomical heterogeneity, and deformation during respiration. These factors may compromise the robustness and reproducibility of optimized plans, especially under ultra-short FLASH delivery times. Moreover, current implementations of SDDRO assume idealized conditions with static targets, which are difficult to replicate in clinical lung settings. To address respiratory motion, breath-hold and respiratory gating have been proposed. Breath-hold offers a reproducible target position and potentially more stable dose-rate delivery, but patient compliance and the short breath-hold window may limit its feasibility for FLASH fractionation. On the other hand, gating techniques allow beam-on only during specific phases of the breathing cycle, accommodating longer treatment times but introducing challenges for maintaining continuous ultra-high dose rate delivery. Emerging solutions, such as predictive motion modeling, fast beam switching, and 4D-SDDRO integration, may enhance adaptive FLASH planning for mobile lung targets. Nonetheless, rigorous preclinical studies and prospective trials are needed to validate these strategies and fully realize the clinical potential of FLASH-RT in thoracic oncology.

### 3.4 Motion management

Respiratory motion management is a critical aspect of FLASH radiotherapy for lung applications. The rapid breathing motion of the lungs can cause the tumor to move significantly during the short FLASH treatment time, potentially leading to inaccurate dose delivery and increased normal tissue damage.

One of the commonly used motion management techniques in CONV-RT for the lungs is breath-hold techniques. In deep inspiration breath-hold (DIBH) or deep expiration breath-hold (DEBH) techniques, the patient is coached to hold their breath at a specific phase of the respiratory cycle. This reduces the motion of the lungs and the tumor, allowing for more accurate radiation delivery (135–137, 150–152). Gating techniques are also widely used in CONV-RT for motion management. In gating, the radiation beam is only turned on when the tumor is within a predefined target volume. This is achieved by synchronizing the radiation delivery with the respiratory motion of the patient, which is monitored using external surrogates such as respiratory belts or internal markers such as implanted fiducial markers (153–155). A study conducted by Yunjie Yang et al. revealed that in silico simulations and phantom measurements were utilized to explore the impacts of respiratory motion on the delivered dose in proton pencil-beam scanning (PBS) transmission FLASH-RT. In comparison to static delivery, motion-induced dose degradation manifested in the forms of translation and distortion. Notably, when beam delivery took place at the phase of maximum inhalation or exhalation, the distortion effect was at a minimum. For FLASH-RT scenarios of clinical relevance, taking into account both treatment delivery and respiratory motion characteristics, gated delivery or deep inspiration breath-hold could serve as an effective motion-management strategy (156).

### 3.5 Barriers to clinical implementation

Despite encouraging preclinical results, the clinical adoption of FLASH radiotherapy—especially for thoracic tumors—faces multiple challenges. Current clinical linear accelerators are often incapable of achieving the ultra-high dose rates required, particularly for deep-seated targets like the lungs (157). Real-time dosimetry at sub-millisecond resolution remains underdeveloped, with limited access to validated detectors such as diamond sensors or ultrafast ion chambers. Motion management is another major hurdle. While 4D-CT and respiratory gating offer potential solutions, their integration with FLASH beam delivery is not yet standardized. The mismatch between FLASH’s short irradiation times and respiratory-induced tumor motion increases uncertainty in dose delivery.

Infrastructure limitations also impede clinical rollout. Most centers lack FLASH-capable hardware, quality assurance protocols, and trained personnel. Furthermore, trial design is hampered by the absence of validated pulmonary toxicity endpoints, long-term safety data, and predictive biomarkers such as oxidative stress indicators (41). To reflect these limitations, **Table 1** has been updated to include a “Current Limitations” column summarizing the major technical and clinical barriers for each beam modality.

## 4 Challenges and solutions

### 4.1 Biological mechanisms and influencing factors remain unclear

The biological mechanisms of FLASH radiotherapy are not yet well-understood. In pre-clinical studies, multiple factors need to be considered, including dose rate, radiation source, dose fractionation, total dose, as well as experimental design and efficacy evaluation methods. The dose rate, being a core parameter, is difficult to measure and control accurately, and its definition lacks uniformity. Besides electron beams, the FLASH effect of X-ray and proton irradiation needs further confirmation. The selection of dose fractionation and total dose requires comprehensive consideration of multiple factors. Currently, research focuses more on protecting normal tissues rather than controlling tumors, and the long-term effects are uncertain. Appropriate experimental models and endpoint indicators are needed for efficacy evaluation.

### 4.2 Technical challenges

Measurement and Control: Existing methods cannot accurately, stably, and real-time measure and control ultra-high dose rate radiation. There is a need to develop or improve dose measurement techniques.

Image-Guided and Positioning: FLASH radiotherapy requires highly precise alignment. Currently, there is no high-precision image-guided and positioning system, and traditional techniques cannot meet the requirements. New technologies need to be developed for real-time monitoring and feedback.

Radiation Beam Transmission and Modulation: Special design and optimization are required for the transmission and modulation of radiation beams. Most pre-clinical studies on FLASH radiotherapy use low-energy electron beams, which are only suitable for treating superficial tumors. To treat deep-seated tumors, new devices such as FLASH-high-energy electron, FLASH-X-ray, or FLASH-proton devices need to be developed. However, the power of accelerators for generating FLASH-X-rays or FLASH-protons should be at least 100 times that for generating FLASH-electrons, and the conversion targets for producing photons or protons should have special characteristics to withstand huge instantaneous power, posing significant technical challenges.

Dose Calculation and Optimization: Traditional methods struggle to accurately predict the dose distribution and shape. Current dose calculation methods, such as the Monte Carlo method, semi-empirical formulas, and analytical methods, have their limitations. New dose calculation methods need to be developed, or existing ones need to be improved and calibrated to meet the requirements of FLASH radiotherapy.

Development of Ultra-High Dose Rate Equipment: The dose rate of FLASH radiotherapy is 3–4 orders of magnitude higher than that of traditional radiotherapy, presenting a huge challenge for equipment development. Currently, global FLASH equipment and experiments are mainly focused on proton and electron beams. For X-rays, which are commonly used in radiotherapy, due to the target conversion efficiency issue, it is extremely challenging to achieve the required dose rate, and it is generally considered unlikely by industry experts. Fortunately, China is at the forefront of this technology. Teams from Zhongjiu Flash Medical Technology Co., Ltd., the Institute of Applied Electronics of the China Academy of Engineering Physics have basically overcome this global problem.

FLASH radiotherapy technology shows great potential, but its clinical translation requires overcoming technical challenges. Dose calculation and optimization are crucial issues. With the continuous progress and improvement of technology, FLASH radiotherapy is expected to play an increasingly important role in future radiotherapy.

## 5 Clinical translation

### 5.1 Clinical trial landscape

Since the first-in-human application of FLASH radiotherapy (FLASH-RT) in 2019, several clinical trials have explored its feasibility and safety. Notably, a French team treated a 75-year-old patient with T-cell cutaneous lymphoma using a 5.6-MeV electron beam at >106 Gy/s (15 Gy total dose), resulting in significant tumor regression within 10 days and only mild acute toxicity such as erythema and edema (112). Importantly, no pulmonary complications were observed, as the irradiation site was cutaneous. This case demonstrated the clinical potential of FLASH-RT and laid the groundwork for future trials.

Subsequently, the University of Cincinnati initiated the FAST-01 trial—marking the first human application of FLASH proton therapy. Targeting painful bone metastases in extremities, FLASH-RT was delivered within 0.3 seconds per site. The trial confirmed procedural feasibility, pain relief efficacy, and an acceptable safety profile (158). However, due to the peripheral anatomical sites, the trial did not provide insight into thoracic or pulmonary toxicities. FAST-02, an extension of this program, aims to evaluate FLASH-RT in thoracic skeletal metastases; patient accrual has concluded, with follow-up underway (159).

In Europe, the University of Lausanne launched the IMPULSE Phase I dose-escalation trial to evaluate FLASH-RT in patients with cutaneous melanoma metastases using 9 MeV electron beams (22–34 Gy). Early data presented at ESTRO 2023 showed that acute skin toxicity remained ≤ Grade 2, with no significant long-term adverse events during 12-month follow-up. These findings underscore the feasibility of FLASH-RT in superficial tumors (160). Building on this, the Lausanne team, in collaboration with the FLASH-KNiFE Consortium, announced additional trials targeting intraoperative radiotherapy (IORT) for skin, abdominal, and head-and-neck tumors. These studies aim to assess the scalability, tissue-specific protection, and oncologic efficacy of FLASH-RT in broader clinical scenarios.

Despite these promising developments, clinical translation to pulmonary and deep-seated thoracic tumors remains limited. Multiple factors contribute to this translational bottleneck: Respiratory motion complicates beam delivery at ultra-high dose rates, requiring motion-adaptive strategies such as 4D-CT or respiratory gating that are not yet standardized for FLASH; Dosimetric challenges arise from lung heterogeneity (air-tissue interfaces), necessitating novel UHDR-adapted detectors with sub-millisecond response times (161); Most trials lack pulmonary-specific toxicity endpoints, such as radiation pneumonitis, fibrosis markers, or longitudinal pulmonary function; Biomarker-based patient selection, such as circulating oxidative stress markers (e.g., MDA, 8-OHdG), remains speculative and has not been integrated into trial designs.

To facilitate FLASH-RT’s application in lung cancer, future clinical trials should incorporate thoracic-specific protocols, dose- and volume-adapted constraints, and translational endpoints (e.g., lung imaging, immune signatures, serum markers of fibrosis).

## 6 Future directions

### 6.1 Technological optimization and equipment upgrades

Future optimization of FLASH radiotherapy systems must address key technical challenges that currently hinder their widespread clinical application, especially in deep-seated or thoracic tumors. Improving the spatial and temporal precision of UHDR delivery systems is critical to minimizing dose heterogeneity, particularly in the context of respiratory-induced tumor motion. Integration of real-time beam gating, 4D-CT planning, and motion-synchronized delivery protocols remains a high priority. Moreover, advancements in dosimetry—such as the development of sub-millisecond response detectors, UHDR-calibrated ion chambers, and FLASH-capable QA systems—are essential for accurate dose verification and regulatory approval.

### 6.2 Pulmonary-specific clinical applications

Despite promising preclinical findings, the translation of FLASH-RT to thoracic malignancies remains limited. Unique anatomic and physiological challenges—such as lung tissue heterogeneity, air-tissue interfaces, and susceptibility to radiation-induced pneumonitis—necessitate tailored approaches. Standardization of pulmonary-specific endpoints, including radiographic fibrosis scoring, pulmonary function testing, and validated serum biomarkers (e.g., MDA, 8-OHdG), will enable better assessment of clinical efficacy and safety. Future trials should incorporate stratified patient recruitment based on baseline pulmonary function and radiation risk to ensure meaningful outcome measures.

### 6.3 Integrative treatment strategies

The combination of FLASH-RT with systemic therapies, including immune checkpoint inhibitors and DNA repair-targeting agents, represents a promising strategy to enhance therapeutic efficacy while mitigating toxicity. Preclinical evidence suggests FLASH may preserve immune cell viability while enhancing CD8^+^ T-cell infiltration in some contexts, raising the possibility of synergy with immunotherapy. Rational trial design should explore optimal sequencing, dose fractionation, and potential biomarkers to guide patient selection in these combined modalities.

### 6.4 Mechanistic and biomarker-driven research

Further elucidation of the underlying biology of the FLASH effect remains essential. Investigations into mitochondrial metabolism, redox homeostasis, DNA damage repair kinetics, and immune modulation will inform biomarker development and patient stratification. Long-term toxicity data, especially regarding late-onset fibrosis and second malignancies, are currently lacking and should be prioritized through well-designed longitudinal studies. Incorporating omics approaches and machine learning may facilitate predictive modeling of individual responses to FLASH.

### 6.5 Interdisciplinary collaboration

Translating FLASH-RT into routine clinical practice will require seamless coordination among diverse disciplines. Medical physicists must develop validated real-time dosimetry and QA protocols tailored to UHDR environments. Bioengineers will be essential for designing scalable, miniaturized linear accelerators or proton systems suitable for lung targets. Clinical trialists must construct multicenter studies with pulmonary endpoints, while regulators and policymakers define technical standards and toxicity thresholds specific to FLASH. The integration of engineering, physics, oncology, and informatics expertise will be pivotal to surmounting translational barriers.

In summary, the successful clinical integration of FLASH radiotherapy—particularly for thoracic applications—will depend on a multifaceted strategy encompassing equipment innovation, lung-specific trial design, mechanistic biomarker discovery, and collaborative clinical infrastructure. With continued scientific and regulatory progress, FLASH-RT has the potential to transform radiotherapy paradigms for high-risk thoracic malignancies.

## 7 Conclusion

FLASH-RT represents a paradigm shift in mitigating RILI, offering the potential to enhance therapeutic ratios through ultra-high dose rate delivery and unique radiobiological mechanisms. This review has addressed a critical gap in the literature by focusing specifically on the pulmonary protection conferred by FLASH-RT—an area underrepresented in previous reviews. By integrating recent (post-2023) preclinical findings and early-phase clinical updates, we have highlighted the multifactorial mechanisms involved in FLASH-induced lung sparing, including modulation of oxidative stress, immune responses, and DNA repair pathways.

Despite promising outcomes, challenges remain in dosimetry standardization, beam characterization, and the translation of preclinical lung models to clinical settings. The complexity of lung tissue heterogeneity further complicates the optimization of FLASH parameters. To address these gaps, future research should prioritize the development of lung-specific dose-response models, validation of predictive biomarkers, and novel delivery systems suitable for deep thoracic structures.

With continued interdisciplinary collaboration, FLASH-RT has the potential to redefine the standards of thoracic radiotherapy and provide safer, more effective treatments for millions of patients worldwide.
